# IL-4 Suppresses the Responses to TLR7 and TLR9 Stimulation and Increases the Permissiveness to Retroviral Infection of Murine Conventional Dendritic Cells

**DOI:** 10.1371/journal.pone.0087668

**Published:** 2014-01-29

**Authors:** Uma Sriram, Jun Xu, Robert W. Chain, Linda Varghese, Marita Chakhtoura, Heather L. Bennett, Philip W. Zoltick, Stefania Gallucci

**Affiliations:** 1 Laboratory of Dendritic Cell Biology, Department of Microbiology and Immunology, Temple University, School of Medicine, Philadelphia, Pennsylvania, United States of America; 2 Joseph Stokes, Jr. Research Institute, Division of Rheumatology, Department of Pediatrics, The Children's Hospital of Philadelphia, Philadelphia, Pennsylvania, United States of America; 3 Department of Surgery, The Children's Center for Fetal Research, The Children's Hospital of Philadelphia, Philadelphia, Pennsylvania, United States of America; National Jewish Health and University of Colorado School of Medicine, United States of America

## Abstract

Th2-inducing pathological conditions such as parasitic diseases increase susceptibility to viral infections through yet unclear mechanisms. We have previously reported that IL-4, a pivotal Th2 cytokine, suppresses the response of murine bone-marrow-derived conventional dendritic cells (cDCs) and splenic DCs to Type I interferons (IFNs). Here, we analyzed cDC responses to TLR7 and TLR9 ligands, R848 and CpGs, respectively. We found that IL-4 suppressed the gene expression of IFNβ and IFN-responsive genes (IRGs) upon TLR7 and TLR9 stimulation. IL-4 also inhibited IFN-dependent MHC Class I expression and amplification of IFN signaling pathways triggered upon TLR stimulation, as indicated by the suppression of IRF7 and STAT2. Moreover, IL-4 suppressed TLR7- and TLR9-induced cDC production of pro-inflammatory cytokines such as TNFα, IL-12p70 and IL-6 by inhibiting IFN-dependent and NFκB-dependent responses. IL-4 similarly suppressed TLR responses in splenic DCs. IL-4 inhibition of IRGs and pro-inflammatory cytokine production upon TLR7 and TLR9 stimulation was STAT6-dependent, since DCs from STAT6-KO mice were resistant to the IL-4 suppression. Analysis of SOCS molecules (SOCS1, −2 and −3) showed that IL-4 induces SOCS1 and SOCS2 in a STAT6 dependent manner and suggest that IL-4 suppression could be mediated by SOCS molecules, in particular SOCS2. IL-4 also decreased the IFN response and increased permissiveness to viral infection of cDCs exposed to a HIV-based lentivirus. Our results indicate that IL-4 modulates and counteracts pro-inflammatory stimulation induced by TLR7 and TLR9 and it may negatively affect responses against viruses and intracellular parasites.

## Introduction

Dendritic cells (DCs), sentinels of the immune system and professional antigen presenting cells, are critical to link innate and adaptive immunity [1]. DCs express pathogen and damage sensors, such as TLRs, that trigger expression of co-stimulatory molecules and inflammatory cytokines, which enable DCs to stimulate adaptive immunity [2], [3]. One of the most important consequences of TLR activation is the induction of type I Interferons (IFNs), a family of cytokines pivotal for antiviral immunity [4] and often pathogenic in autoimmunity [5]. For this induction, TLR3 and TLR4 activate the signaling adaptor TRIF, inducing phosphorylation and translocation of IRF3, while TLR7 and TLR9 use a pathway that involves the transcription factor IRF7 and takes place downstream of the MyD88 signaling pathway [6], [7].

TLR7 was initially identified as a receptor able to recognize imidazoquinoline derivatives with antiviral activity, such as imiquimod and resiquimod (R848), and guanine analogues such as loxoribine [8], [9]. Subsequently, guanosine- or uridine-rich single-stranded (ss) RNAs, such as those derived from the human immunodeficiency virus (HIV) and the influenza virus, were identified as natural ligands for TLR7 [10], [11].

TLR9 recognizes unmethylated deoxyribo (cytidine-phosphateguanosine) (CpG) DNA motifs that are present at high frequencies in pathogens such as viruses and bacteria [12].

TLR7 and TLR9 participate in the resistance to many RNA and DNA viruses [13], [14], in the control of endogenous and exogenous retroviruses [15] and TLR7 is involved in HIV recognition [16]. TLR7 and TLR9 are expressed by plasmacytoid DCs but also by other immune cells such as alveolar macrophages and conventional DCs (cDCs) [2], [17]. In particular, cDCs that are generated in culture from mouse bone marrow in the presence of GM-CSF constitutively express TLR7 and TLR9 as well as high levels of IRF7, the master regulator of type I IFNs. Hence, GM-CSF- bone marrow-derived cDCs are ready to respond to viral ligands by producing both IFNα and IFNβ [18] thus making these cells a good experimental model to study innate responses to viruses.

As major sentinels, DCs are strongly influenced by the surrounding environment. The polarization of T cell cytokine production into the Th1 and Th2 patterns has become a basic laboratory manipulation for cellular immunologists, especially in the context of modulating DCs to evoke responses for various immune strategies, *i.e.* vaccination [19]. In spite of the vast literature about the balance of Th1/Th2 responses in homeostasis and disease, many immune consequences of this interplay, competition and mutual inhibition still need investigation. An important example is the lack of clear mechanisms to explain the observed increased susceptibility to viral infections occurring during Th2-inducing pathological conditions such as chronic asthma [20], [21], [22] or Th2-inducing parasitic diseases (*e.g. Schistosoma*) [23], [24], [25]. The co-infection of Human immunodeficiency virus (HIV) and helminth parasites, such as *Schistosoma* spp, is dramatically frequent in sub-Saharan Africa and it adversely impacts the ability of the immune system to control HIV progression [26], [27]. It has been shown that IL-4, a prototype Th2 cytokine, increases Simian immunodeficiency virus load in infected rhesus macaques, despite enhanced antibody responses [26]. The molecular mechanism of these interactions is not fully understood.

Mouse bone marrow-derived cDCs are sometimes generated *in vitro* in the presence of IL-4 because they respond well to some danger signals such as LPS [28], [29], although this is not the case for other stimuli. In fact, we have previously shown that IL-4 suppresses cDC responses to Type I IFNs (both IFNα and IFNβ) [29]. In the present study, we show that IL-4 inhibits the response of cDCs to ligands of TLR7 and TLR9. This inhibition affects both Type I IFN-dependent and NFκB-dependent responses, and it requires the canonical signaling pathway downstream of IL-4 receptor, mediated by STAT6 [30]. Furthermore, IL-4 induces the up-regulation of the SOCS (suppressors of cytokine signaling) molecules, especially SOCS2, that are known to be negative regulators downstream of cytokines [31], [32], [33] and may mediate IL-4 suppressive effects.

Using a HIV-derived lentivirus infection model, we also show that IL-4 decreases DC production of Type I IFN and IFN-responsive genes (IRGs) and increases the permissiveness of DCs to viral infection. Our results provide a molecular explanation for the inhibitory effects of Th2-inducing infections/infestations on the ability of the immune system to mount effective anti-viral responses.

## Materials and Methods

### Ethics statement

Protocols for the use of animals were in accordance with the guidelines of and were approved by the Institutional Animal Care and Use Committees of the Children's Hospital of Philadelphia and of Temple University, both of which are American Association for the Accreditation of Laboratory Animal Care-accredited facilities.

### Mice

C57BL/6, C57BL/6-RAG-1-knock out (RAG-KO), and IFNAR1-KO [34] mice were bred and maintained in our colony and STAT6-KO mice were bought from Jackson Laboratory and used directly in our experiments. Female mice were used between 6 and 14 weeks of age for most of the experiments.

### In vitro cDCs

Mouse bone marrow-derived cDCs were generated as described previously [18], [29]. Briefly, bone marrow precursors from C57BL/6-RAG-KO mice were seeded at 1×10^6^/ml in complete IMDM (10% FBS, penicillin/streptomycin, gentamicin, and β-mercaptoethanol) enriched with 3.3 ng/ml GM-CSF (BD Biosciences) in 24-well plates. One milliliter of medium was added on day 3 and half of the medium was replaced on day 5 and subsequently each day until the culture was used (day 6 or 7). Generating DCs from RAG-KO bone marrow does not require depletion of T and B cells, and RAG-KO cDCs behave identically as those from normal mice. CDCs from STAT6-KO or IFNAR1-KO mice and their wild type C57BL/6 mice were generated from bone marrow precursors that were depleted with anti-CD19, Thy 1.2 and MHC Class II magnetic beads (Miltenyi Biotech) to remove the B cells, T cells and other activated cells, as described elsewhere [18]. Resting DC cultures at day 6 or 7 were treated for 24 h with recombinant mouse IL-4 at 2.5 ng/ml (BD Biosciences), a dose we have previously found efficient to affect cDCs [29]. Without washing out IL-4, we stimulated cells with either 10 ug/ml CpG-B 1826 (synthesized from IDT biotechnologies) or 1 ug/ml R848 (Invivogen). In the experiments to test the IL-4 effects on NF-κB pathway, we treated cells with IL-4 for 24 h or not and then treated with Bay11 (10^−6^ M) for 20 min and then treated with CpG or R848. CDCs were harvested after 6 h stimulation for RNA analysis, 8 h for Western blot analysis and 24 h or 48 h for FACS analysis of surface activation markers, respectively. Culture supernatants were also collected at 6 h, 24 h or 48 h for cytokine analysis by ELISA.

### Flow cytometry

CDCs were washed in cold PBS, incubated with rat anti-mouse CD16/CD32 (clone 2.4G2) mAb for 10 min to block FcγR, and then stained for 30 min on ice with the allophycocyanin-conjugated hamster anti-mouse CD11c and FITC-conjugated mouse anti-mouse H2Kb mAbs (BD Biosciences). Cells were fixed in 1% formaldehyde and analyzed on a FACSCanto cytometer (BD Biosciences). FlowJo software was used for data analysis.

### Immunofluorescence

CDCs were harvested after 24 h treatment with IL-4 and plated onto poly-L-lysine coated slides and incubated at 37°C for 1 h for adherence. Cells were fixed on the slides with 4% paraformaldehyde followed by quick wash in sodium borohydride (0.1% in PBS) for 3 min and blocked for 1 h with blocking buffer containing 0.3 M glycine, 1% BSA, 0.1% Tween 20 and 10% fetal bovine serum in PBS. The slides were stained with rabbit anti-mouse TLR7 (Imgenex, Cat. No. IMG 581-A) overnight at 4°C. The slides were then washed and goat anti-rabbit Cy3 secondary antibody was added for 1 h. Slides were washed and stained with DAPI for 10 min and mounted and analyzed with a fluorescence microscope (Olympus BX60 microscope with Diagnostics Instruments Camera and Spot Advanced Software). At least five fields from each slide were analyzed and the image intensity was calculated using the NIH ImageJ software.

### Stimulation of splenic DCs ex vivo

Spleens from C57BL/6 mice were harvested and incubated in medium with collagenase/DNase for 45 min as described previously [29]. Single cell suspensions were made using 100micron cell strainer; RBCs were lysed, cells were washed, and total spleen cells were plated as equal cell numbers (30–50 million each) in 6-well plate in 3 ml medium with 3.3 ng/ml GM-CSF alone or GM-CSF plus 2.5 ng/ml IL-4 overnight and then stimulated with 50 ug CpG-B 1826 or 5 ug R848 for 4 h at 37°C. Cells were then harvested, washed and sorted by magnetic beads (Miltenyi Biotech) first removing CD19 positive cells and then positively selecting for CD11c labeled cells from the CD19 negative fraction. The CD11c purity was >90% as assessed by flow cytometry (data not shown). The sorted cells were resuspended in TRIzol reagent for RNA extraction.

### Quantitative RT-PCR

Gene expression in cDCs was analyzed by quantitative real-time RT-PCR (qPCR) using Taqman probes. Briefly, RNA was extracted by TRIzol method and repurified using Qiagen columns (Qiagen Inc. USA). CDNA was synthesized using the cDNA archive kit followed by a preamplification reaction (Applied Biosystems). Premade TaqMan primers and probes from Applied Biosystems were used to study the expression of IFN responsive genes (IFNβ, IRF7, ISG15, Mx-1, Oas-3), cytokines (TNFα, IL-12p35, IL-12p40 and IL-6), TLR7, TLR9 and SOCS1, SOCS2 and SOCS3 gene expression. Cyclophilin was used as the reference gene for normalization. The Ct method of relative quantification of gene expression was used for these TaqMan PCRs, and the normalized Ct values (against cyclophilin) were calibrated against the control sample (untreated cDCs) in each experiment [35].

### ELISA

ELISA kits (BD Pharmingen) were used to measure the levels of TNFα, IL-12p70 and IL-6 in the supernatants of cDC cultures stimulated for 6, 24 or 48 h with TLR ligands or medium alone.

### Western blot analysis

Cell lysates from DCs were prepared as previously described[18]. Western blot analyses were performed using total DC cell protein of 30–50 µg for STAT1/2 and SOCS-1,-2,-3 detection and 70 ug for TLR7 detection. In brief, protein samples were denatured by boiling for 5 minutes and loaded onto 10% Bis-Tris gels. After electrophoresis, proteins were transferred to PVDF membranes. Membranes were blocked for 1 h with blocking buffer (2% non-fat milk in PBS), then incubated overnight at 4°C with the primary antibodies diluted in blocking buffer with 0.1% Tween 20 simultaneously: rabbit polyclonal antibodies were used to analyze STAT1 and STAT2 (Upstate Biotechnology, Cat. Nos. 06-501 and 07-140, respectively), IRF7 (Santa Cruz Biotech., CA, USA, Cat. No. sc-9083), and TLR7 proteins (Hycult Biotechnologies, Cat. No. Hp9040) and SOCS 1 (Cat. No. 3950), SOCS2 (Cat. No. 2779) and SOCS3 (Cat. No. 2923) from Cell Signaling Technologies. Mouse anti-GAPDH antibody (Santa-Cruz Biotech) was used as a loading control. After incubating with primary antibodies, the membranes were washed with PBS containing 0.1% Tween 20 (PBST) three times. Then the membranes were incubated for 1 h with IR Dye 800 goat anti-rabbit and IR Dye 680 goat anti-mouse (LI-COR Biosciences) diluted in blocking buffer plus 0.1% Tween 20. The blots were then washed three times with PBST and rinsed with PBS. Proteins were visualized by scanning the membrane on an Odyssey Infrared Imaging System (LI-COR Biosciences) in both 700 nm and 800 nm channels.

### Lentiviral production

HIV-1-based lentiviruses were produced as previously described [36]. Briefly, the GFP reporter under the transcriptional control of the CMV promoter was inserted into the previously described lentiviral transfer plasmid [36]. Single cycle self-inactivating HIV-1-based vectors, pseudotyped with the vesicular stomatitis-G protein envelop, were generated by three-plasmid co-transfection in HEK293-T cells [37]. Viral supernatants, concentrated by ultracentrifugation, were titrated using serial dilutions.

### Viral infection in cDCs

CDCs from C57BL/6 mice were grown as described above in GM-CSF-enriched complete medium with or without recombinant mouse IL-4 for 6 days and then were infected with the self-inactivating HIV-1 based lentivirus expressing a GFP reporter as described above at a Multiplicity of infection (MOI) of 10 for 5 h and then harvested for RNA extraction and analyzed for IFN responsive gene expression (IRF7, ISG15 and Oas-3) by real-time RT PCR. In a separate set of experiments, cDCs, grown with or without recombinant mouse IL-4, were infected at day 2 with the same lentivirus at MOI of 5 and 10 and then harvested at day 6 and analyzed for GFP positivity by flow cytometry (BD FACS Canto) to determine efficiency of infection. The infection was optimum at 96 h and we tested the IL-4 effects at this time point in all experiments.

### Statistical analysis

Data were analyzed using Prism software (GraphPad, San Diego). One sample t-test or two-tailed Student's *t* test, as appropriate, was used as statistical test for the different sets of experiments, and considered significant values of *p*<0.05 (marked in the figures as *p<0.05; **p<0.01; *** *p*<0.001).

## Results

### IL-4 suppresses the expression of IFN-responsive genes in cDCs upon TLR7 and TLR9 stimulation

An important consequence of TLR7 and TLR9 stimulation is the induction of Type I IFNs and IRGs, including many anti-viral genes [9], [12]. Type I IFNs, through their receptor IFNAR, create an autocrine feedback loop to sustain their signaling and amplify their stimulation [38], [39]. We have previously shown that IL-4 suppresses the response of cDCs to Type I IFNs *in vitro* and *in vivo* [29]. In the present study, we analyzed the effects of exposure to IL-4 on the response of cDCs to TLR7 or TLR9 stimulation, first measuring the expression levels of IRGs. We treated cDCs for 24 hours with or without 2.5 ng/ml of IL-4 before stimulating them with the TLR7 ligand R848 or TLR9 ligand CpGs for 6 hours. IL-4 suppressed the up-regulation of ISG15 and Mx-1.

**Figure 1:**
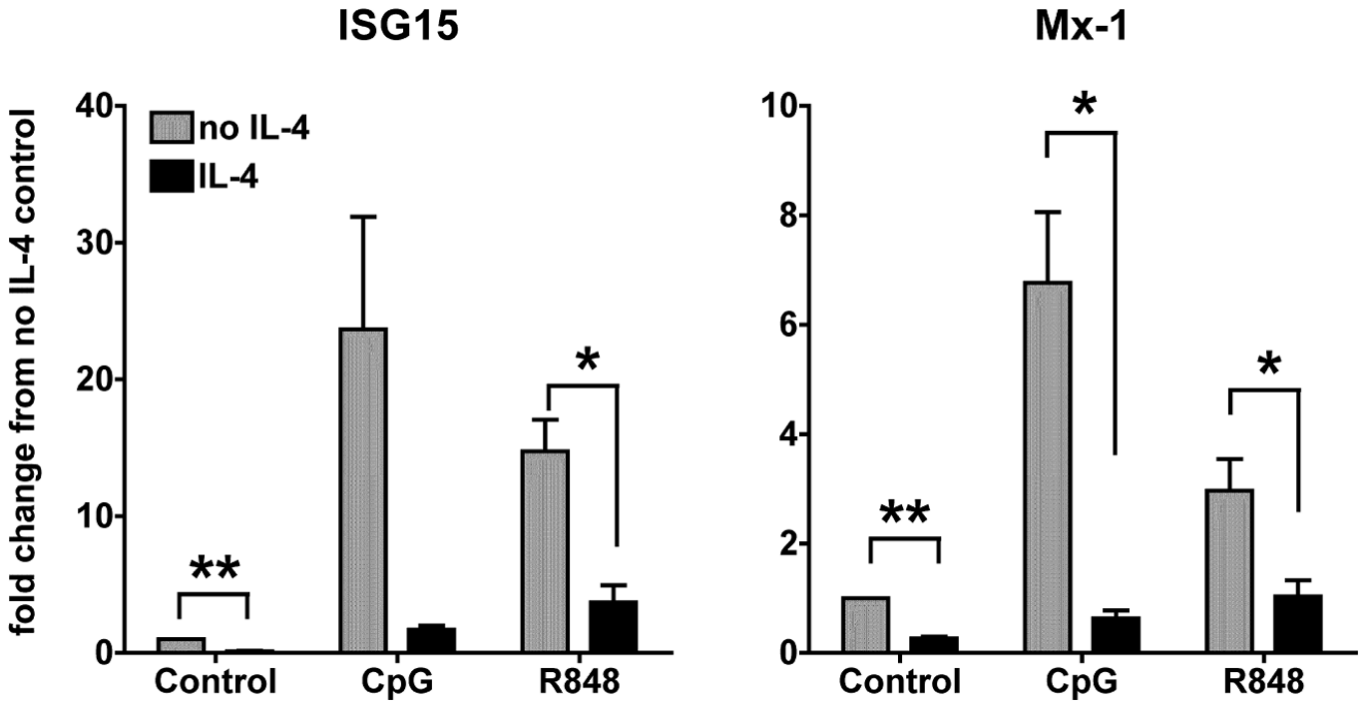
IL-4 suppresses the expression of TLR7- and 9-induced IFN-responsive gene expression. We treated cDCs at day 6-7 of culture with IL-4 or left them untreated for 24 h. We then stimulated the cDCs with CpG 1826 (10 ug/ml) or R848 (1 ug/ml) for 6 h and then analyzed by qPCR the expression of the IFN-responsive genes ISG15 and Mx-1. All of the conditions were normalized against the control (untreated DCs in medium only) in each experiment. Results are average of three independent experiments, performed with three independent bone marrow-derived cultures from 3 different mice; *p<0.05; **p<0.01.

Interferon-stimulated gene 15/ubiquitin cross-reacting protein (designated ISG15/UCRP) is a 15-kDa ubiquitin-like protein identified as a product of an IFN-stimulated gene in humans [40]. ISG15 expression is induced in many cell types by IFNs, viral infection, bacterial endotoxins, and genotoxic stress [41]. We found that R848 and CpG induced a 15–20 fold increase of ISG15 gene expression in cDCs, measured by quantitative real time RT-PCR (qPCR), which IL-4 strongly reduced (Figure 1). The suppression of the CpG-induced ISG15 was not statistically significant. This was due to variation in the CpG-induced ISG15 up-regulation between experiments, while the suppression was very consistent.

Mx-1 is an important anti-viral gene [42], which is induced upon TLR7 and TLR9 stimulation in cDCs (3 and 7 fold increase with R848 and CpG, respectively), and was significantly suppressed by IL-4 (Figure 1).

We also found that cDCs treated with IL-4 expressed lower levels of IFNβ, measured as gene expression by quantitative real time RT-PCR (qPCR), than cDCs that were not exposed to IL-4 (Figure S1). Although we observed a strong trend of inhibition, the differences did not reach statistical significance because of variations between experiments. Since IFNβ is an early gene, expressed rapidly 1 hour after TLR stimulation, we also measured this early expression and found the same trend of inhibition of such early expression of IFNβ (data not shown), suggesting a direct suppression of the signaling pathway downstream of TLR7 and TLR9.

Our results indicate that IL-4 inhibits the up-regulation of anti-viral genes in cDCs upon TLR7 and TLR9 stimulation (Figures 1 and S1).

### IL-4 suppresses the expression of IRF7 in cDCs upon TLR7 and TLR9 stimulation

**Figure 2:**
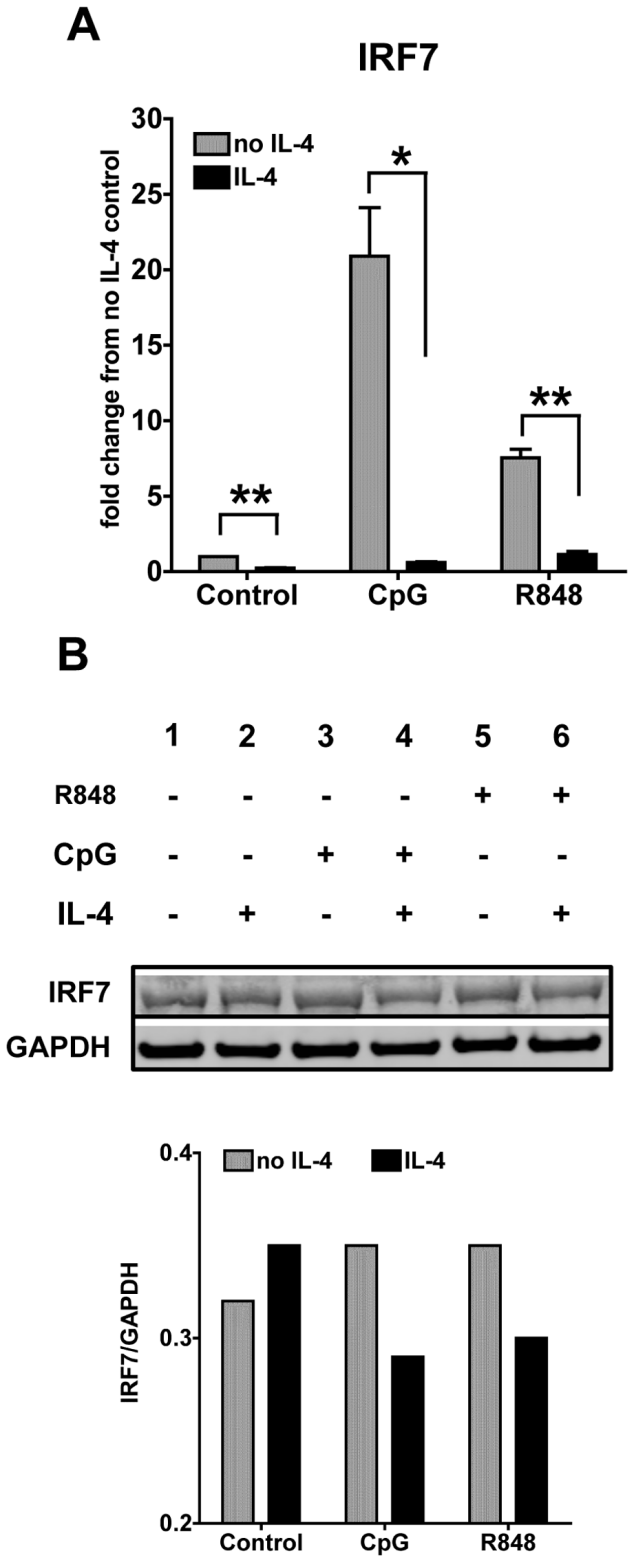
IL-4 suppresses the expression of TLR7- and TLR9-induced IRF7 expression. **A.** We analyzed the gene expression of IRF7 by qPCR after stimulation with CpG 1826 (10 ug/ml) or R848 (1 ug/ml) for 6 h in cDCs treated with IL-4 or left untreated for 24 h. All of the conditions were normalized against the control (untreated DCs in medium only) in each experiment. Results are the average of three independent experiments; *p<0.05; **p<0.01. **B.** We analyzed the IRF7 protein expression by Western blotting in cDCs 8 h after stimulation with CpG or R848. Bar graph represents the ratio of integrated density values of IRF7 vs GAPDH (loading control). One representative blot is shown from testing five independent cultures.

IRF7 is one of the most important signaling molecules downstream of TLR7 and TLR9 and it is the master regulator of the autocrine feedback loop fueling the production of Type I IFNs and the anti-viral response [43], [44]. We found that stimulation of TLR7 and TLR9 induces a strong up-regulation of IRF7 gene expression, with CpGs inducing a more pronounced up-regulation than R848 (20 vs. 7 folds increase, respectively) (Figure 2A). IL-4 treatment significantly suppressed the IRF7 gene expression induced by both ligands (Figure 2A). These results were also confirmed at the protein level. We analyzed by western blot cells lysates of cDCs exposed to IL-4 for 24 hours and then stimulated with TLR ligands for 8 hours and found that IL-4 decreased the levels of IRF7 in cDCs stimulated with CpG and R848 (Figure 2B). This data suggests that the inhibitory effects of IL-4 on the response to TLR7 and TLR9 stimulation are in part mediated by decreasing the autocrine feedback loop through the IFN master regulator IRF7, which normally fuels the production of Type I IFNs and the anti-viral response.

### IL-4 inhibits the up-regulation of MHC Class I induced by TLR7 and TLR9 stimulation

**Figure 3:**
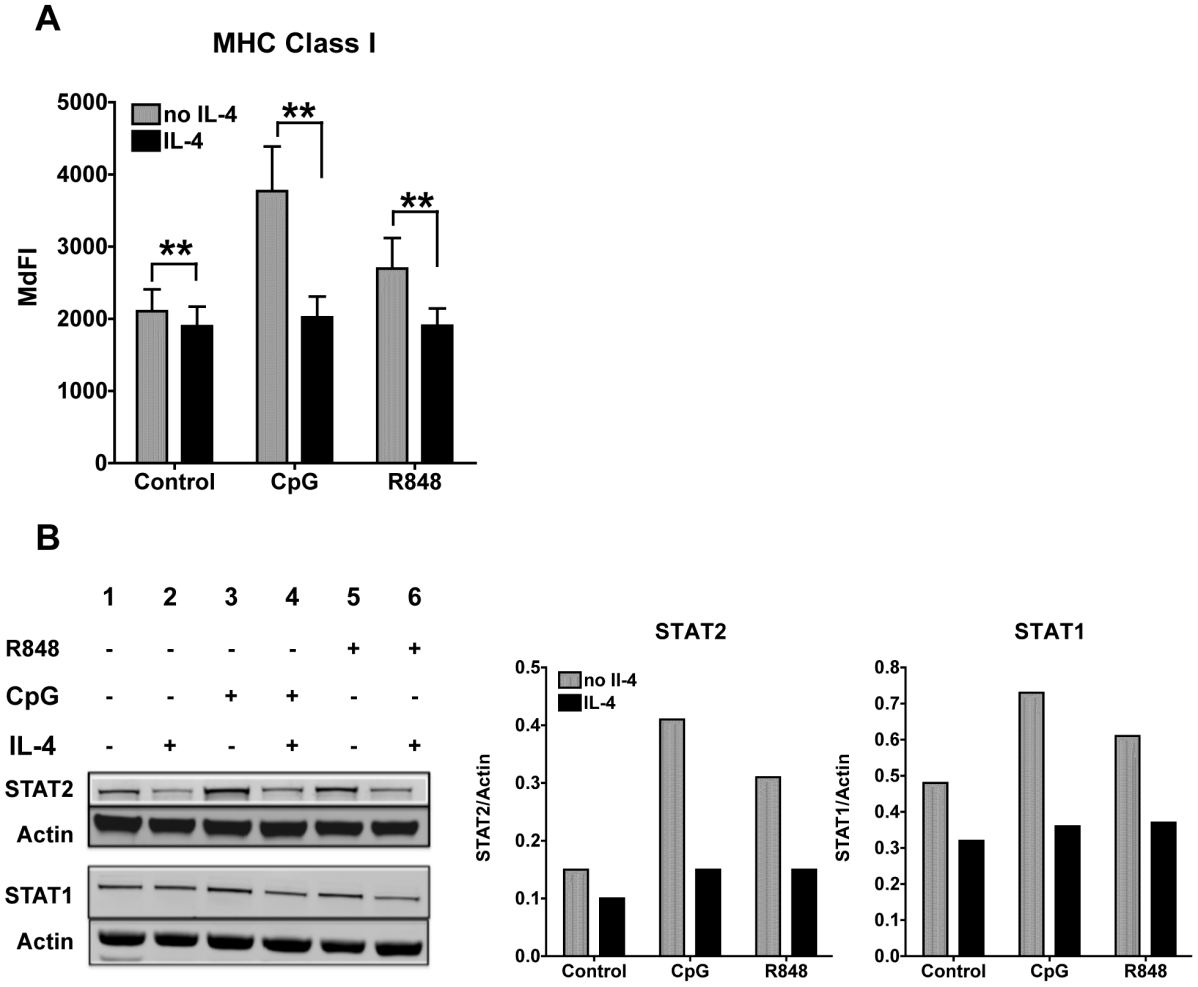
IL-4 suppresses expression of TLR7- and 9-induced MHC Class I, STAT1 and STAT2 protein expression. **A**. We treated cDCs with IL-4 or left them untreated for 24 h. We then stimulated the cDCs with CpG or R848 for 24 h and then analyzed MHC class I expression. Histogram bars represent averages and standard errors (SE) of the median fluorescence intensity (MdFI) of seven experiments conducted with seven independent cDC cultures; *p<0.05,**p<0.01. **B**. Western Blot analyses of STAT2 and STAT1 expression in cDCs treated or not IL-4 for 24 h and then stimulated for 8 h with 10 ug/ml of CpG 1826 or 1 ug/ml of R848. Actin was used as loading control. We show a representative blot of three independent experiments. Bar graph represents the ratio of integrated density values of STAT1 or STAT2 vs Actin (loading control).

We have previously shown that IL-4 suppresses LPS- and Type I IFN-induced MHC Class I surface expression on cDCs [29]. We investigated whether the MHC up-regulation induced by TLR7 and TLR9 stimulation was also affected by IL-4. Indeed, we found that the induction of MHC Class I upon CpG and R848 stimulation was significantly suppressed by IL-4 treatment in cDCs, suggesting that downstream effects of the TLR stimulation are also inhibited by IL-4 (Figure 3A).

### IL-4 suppresses STAT1 and STAT2 expression induced by TLR7 and TLR9 stimulation

TLR7 and TLR9 stimulation induces the production of Type I IFNs that signal through their receptors IFNARs and the downstream signaling molecules STAT1 and STAT2, to induce the transcription of IRGs. STAT1 mediates responses to both IFNα/β and IFNγ, while STAT2 is specific of the signaling pathway downstream of IFNARs. Moreover, STAT1 and STAT2 are IRGs themselves, being up-regulated upon IFNα/β stimulation [29]. Therefore, to better understand the effects of IL-4 on the autocrine feedback loop of Type I IFNs that amplifies the responses to TLR7 and TLR9, we assessed whether IL-4 treatment affected the protein levels of STAT1 and STAT2. STAT1 and STAT2 are constitutively expressed in cDCs and 8 hours of stimulation with both CpG and R848 increased STAT levels, STAT2 more than STAT1, as shown by Western Blot analysis (Figure 3B). IL-4 greatly suppressed the up-regulation of STAT1 and STAT2 induced by both TLR ligands (Figure 3B). These results are in concordance with our previous report showing that IL-4 suppresses STAT expression induced by IFN α/β [29]. These findings support the hypothesis that the suppressive effects of IL-4 on TLR7/9 responses are in part mediated by the inhibition of the IFN signaling pathway. Moreover, we found that the gene expression of IFNAR1 and IFNAR2 was also suppressed by IL-4 (Figure S3), further validating this hypothesis.

### IL-4 suppresses the expression and production of IL-6, TNFα and IL-12 by cDCs upon TLR7 and TLR9 stimulation

**Figure 4:**
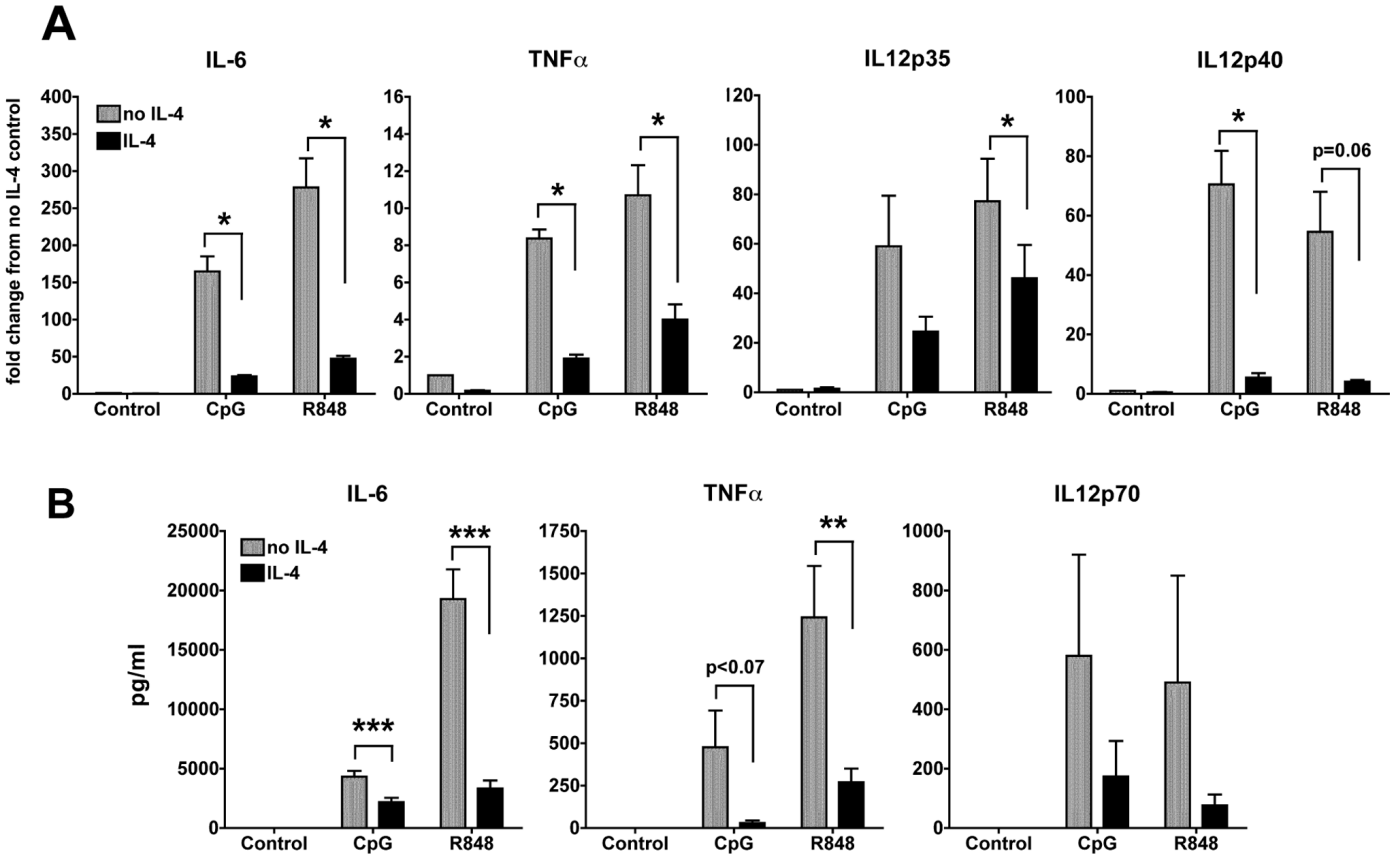
IL-4 suppresses TLR7- and TLR9-induced production of pro-inflammatory cytokines. **A.** We analyzed the gene expression of IL-6, TNFα, IL-12p35 and IL-12p40 by qPCR after stimulation with CpG or R848 for 6 h in cDCs treated or not with IL-4 for 24 h. Averages and SE of four independent cDC cultures are shown (*p<0.05; ** *p*<0.01, ***p<0.001). **B.** We measured by ELISA the levels of IL-6, TNFα and IL-12p70 in the supernatants of DC cultures treated or not with IL-4 for 24 h and then stimulated with R848 or CpG 1826 for 24 h. Averages and SE of four independent cultures are shown.

In both humans and mice, stimulation of TLR7 and TLR9 results in vigorous production of multiple pro-inflammatory cytokines such as IL-6, IL-12 and TNFα [45]. We have shown in a previous report that IL-6 is highly induced by Type I IFN stimulation, particularly by IFNβ and that IL-4 inhibits IL-6 production [29]. We analyzed the gene expression of IL-6, the two subunits of IL-12, IL-12p35 and IL-12p40, as well as TNFα in cDCs that were treated or not with IL-4 for 24 hours and then stimulated with CpG or R848 for 6 hours. IL-4 significantly down-regulated the gene expression of IL-6, IL-12, and TNFα. The suppression of CpG-induced IL-12p35 and R848-induced IL-12p40 did not reach statistical significance because of variation in the levels of induction by the TLR ligands, while the suppression was consistent in all cases (Figure 4A). Protein production and secretion of these cytokines, measured by ELISA, also followed the same trend as the gene expression (Figure 4B). The cDCs did not secrete detectable levels of pro-inflammatory cytokines in absence of stimulation, indicating that we are generating truly resting DCs with no pro-inflammatory activity [46]. Stimulation with CpG and R848 induced high levels of IL-6 (3 ng/ml and up to 20 ng/ml, respectively) and IL-4 significantly inhibited this production (Figure 4B). CDCs also produced IL-12p70 and TNFα upon CpG and R848 stimulation, and IL-4 suppressed the secretion of these cytokines as well (Figure 4B).

In order to study the signaling pathways that are affected by IL-4, we blocked two major pathways downstream of TLR7/9, Type I IFNs and NF-κB, using cDCs generated from IFNAR1-KO mice and treatment of the cDCs with Bay11, an inhibitor of NF-κB activation [47]. We treated WT and IFNAR1-KO cDCs with or without IL-4 for 24 hours. Where indicated, cells were treated with Bay11 for 20 min prior to the addition of CpG or R848 for 24 hours. In this way, cDCs were exposed to IL-4 for a total of 48 hours, to Bay11 for 24 hours and 20 minutes, and to TLR ligands for 24 hours. We found that production of IL-12 and TNFα upon activation of cDCs with CpG or R848 was partly IFN- and partly NFκB-dependent (Figure S2). In the two independent cultures that we have tested (Figure S2), we found a clear trend of IL-4 strongly suppressing both IL-12p70 and TNFα induced by CpG (data not shown) and R848 (Figure S2), suggesting that IL-4 suppression of these cytokines is probably affecting both IFN and NFκ-B pathways. IL-6 production was partly IFN-dependent, and IL-4 inhibited an amount of IL-6 equivalent to the IFN-dependent part. Bay-11 did not affect IL-6 and IL-4 could not completely inhibit IL-6 production, suggesting that a distinct signaling pathway leads to IL-6 stimulation, which is IFN- and NF-κB-independent and is not affected by IL-4.

In summary, these results suggest that IL-4 suppresses cDC production of pro-inflammatory cytokines upon TLR7 and TLR9 stimulation by inhibiting both IFN-dependent and NF-κB-dependent responses.

### IL-4 suppression of TLR7- and TLR9-induced activation of cDCs is STAT6- dependent

**Figure 5:**
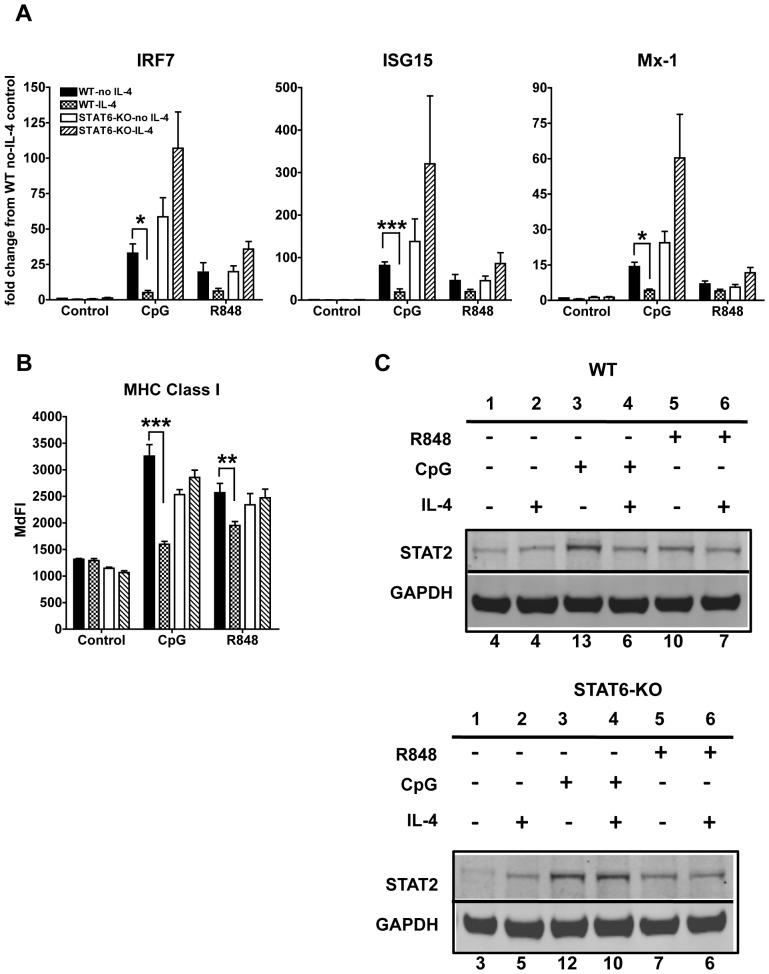
IL-4 suppression of TLR7- and TLR9-induced IFN-dependent response in cDCs is STAT6 dependent. **A.** We analyzed by qPCR the gene expression of the IFN-responsive genes IRF7, ISG15 and Mx-1 induced by 6 h stimulation with CpG or R848 in wild type (WT) (C57BL/6) and STAT6-KO cDCs treated or not for 24 h with IL-4. Results were normalized to WT control cells not treated with IL-4. **B.** We analyzed the MHC Class I expression on cDCs by flow cytometry. Results are shown as median fluorescence intensity (MdFI). Averages and SE of three independent BMDC cultures are shown. (* p<0.05,**p<0.01, ***p<0.0001). **C.** Western Blot analyses of STAT2 expression in cDCs treated or not for 24 h with IL-4 and harvested 8 h after stimulation with CpG or R848 in WT and STAT6-KO BMDCs. GAPDH was used as loading control. One blot representative of three independent experiments is shown. Numbers below the blot represent the percentage of the normalized integrated density values against GAPDH (loading control).

After binding to its receptor, IL-4 primarily signals through STAT6 to bring about its effects [30]. Therefore, to better understand the mechanisms of the anti-inflammatory effects of IL-4, we investigated whether activation of STAT6 is required for the IL-4-mediated inhibition of cDC responses to TLR7 and TLR9 ligands. We tested cDCs generated from STAT6-KO mice in comparison with cDCs from C57BL/6 wild type mice. We found that IL-4 could not suppress the up-regulation of the IFN-responsive genes ISG15 and Mx-1 as well as IRF7 in STAT6-KO cDCs (Figure 5A). On the contrary, treatment with IL-4 actually enhanced the up-regulation of IRGs upon TLR7/9 stimulation in STAT6-KO cDCs, although it did not reach statistical significance (Figure 5A). IL-4 suppression of MHC Class I up-regulation required STAT6 as well; however there was no augmentation of the MHC Class I upon TLR stimulation of STAT6-KO cDCs as observed for the anti-viral genes (Figure 5B). Furthermore, the inhibition of TLR-induced up-regulation of STAT2 mediated by IL-4 was abrogated in STAT6-KO cDCs (Figure 5C). These results indicate that IL-4 inhibition of the IFN autocrine feedback loop, triggered by TLR7/9, requires STAT6.

**Figure 6:**
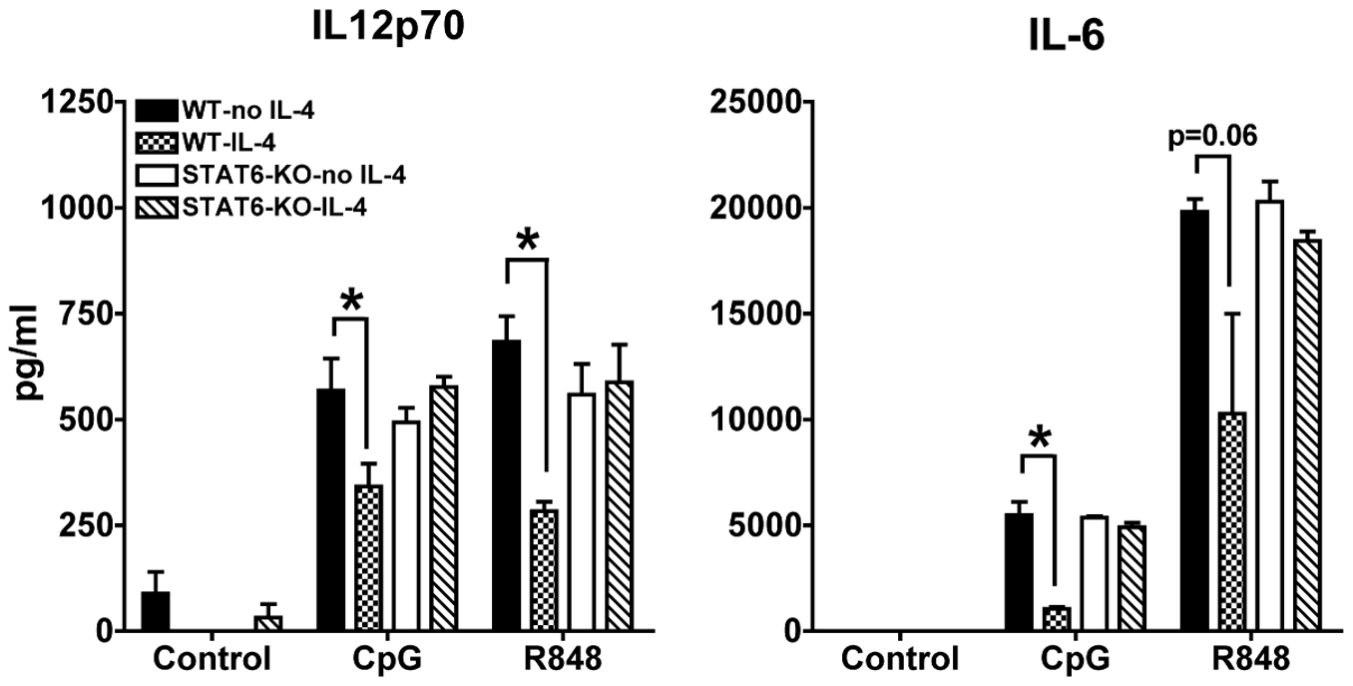
IL-4 suppression of pro-inflammatory cytokines is STAT6-dependent. We measured by ELISA the levels of IL-6 and IL-12p70 in the supernatants of cDC cultures from wild type and STAT6-KO mice treated or not for 24 h with IL-4 and then stimulated with CpG or R848 for 6 h (IL-6) or 48 h (IL-12p70). Averages and SE of three independent cultures are shown (* p<0.05).

In addition, we found that the production of IL-12p70 and IL-6 induced by both TLR7 and TLR9 ligands was inhibited by IL-4 in a STAT6-dependent manner (Figure 6). We observed very low levels of TNFα upon CpG induction in these cultures; however, TNFα induced by R848 was inhibited by IL-4 in a STAT6-dependent way (data not shown).

In summary, our results indicate that IL-4 inhibition of cDC responses to TLR7/9 requires STAT6.

### IL-4 suppresses TLR7 and TLR9 gene but not protein expression in cDCs

**Figure 7:**
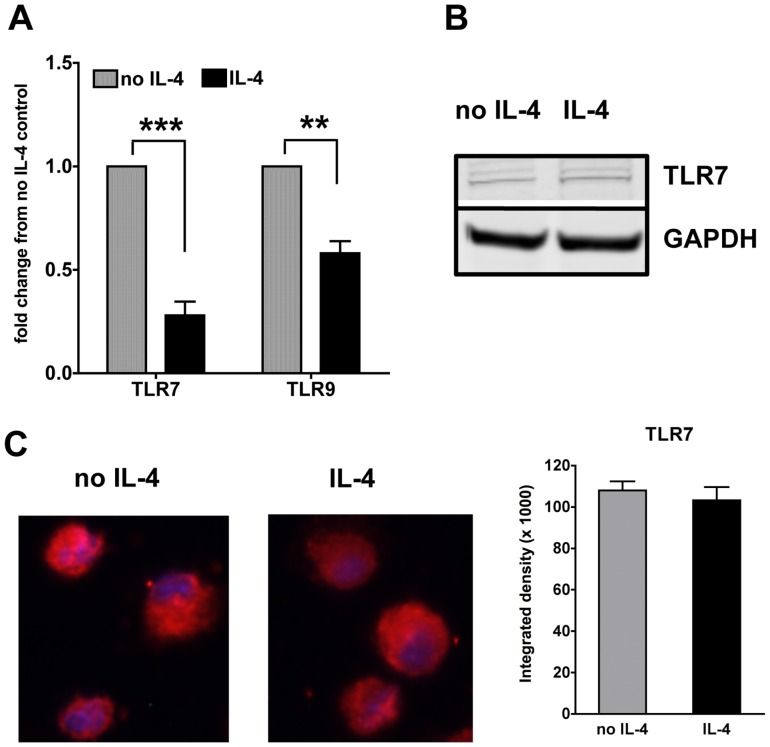
IL-4 suppresses TLR7 and TLR9 at the gene but not the protein level in cDCs. **A.** We analyzed by qPCR the gene expression of TLR7 and TLR9 in cDCs treated or not with IL-4 for 24 h. Graphs show averages and SE of six independent cultures. **B.** We analyzed the protein expression of TLR7 in the total lysates of cDC treated or not for 24 h with IL-4. **C.** We analyzed by immunofluorescence staining TLR7 expression in cDCs after 24 h treatment with IL-4. The nucleus was stained with DAPI and TLR7 was stained using a rabbit polyclonal anti-body to TLR7 and a secondary anti-rabbit IgG conjugated to Cy3. A minimum of five fields in each slide was read for each condition at a magnification of 40×. Picture representative of five independent cultures.

To further understand the mechanism of suppression, we assessed whether IL-4 targets directly the expression of TLR7 and TLR9. So we first studied the gene expression of TLR7 and TLR9 in the IL-4-treated cDCs. Exposure of non-stimulated cDCs to IL-4 for up to 24 hours significantly decreases the gene expression of both TLR7 and TLR9 (Figure 7A). The effect was much more profound with TLR7 than TLR9. Upon TLR stimulation, we observed a significant increase in the TLR7 gene expression with R848 and CpG while TLR9 was slightly decreased by both TLR ligands (Figure S4). IL-4 suppressed the gene expression of both TLRs independently of TLR stimulation (Figure S4). We next tested whether TLR protein levels were decreased in cDCs upon treatment with IL-4 for 24 hours. We could not find a good antibody to measure TLR9 protein expression in cDCs, and indeed most studies on TLR9 expression utilize cells expressing GFP-TLR9 fusion protein [48]. On the contrary, we could easily detect TLR7 with two distinct techniques. To our surprise, we did not see any decrease in the TLR7 protein expression as analyzed by western blot of total cDC lysates (Figure 7B) or by immunofluorescence staining of cDCs (Figure 7C). This suggests that although the gene expression is regulated by IL-4, the overall suppressive effects of IL-4 may not involve TLR7 as the protein levels were not altered.

### IL-4 induces SOCS 1 and 2 gene and protein expression in cDCs

**Figure 8:**
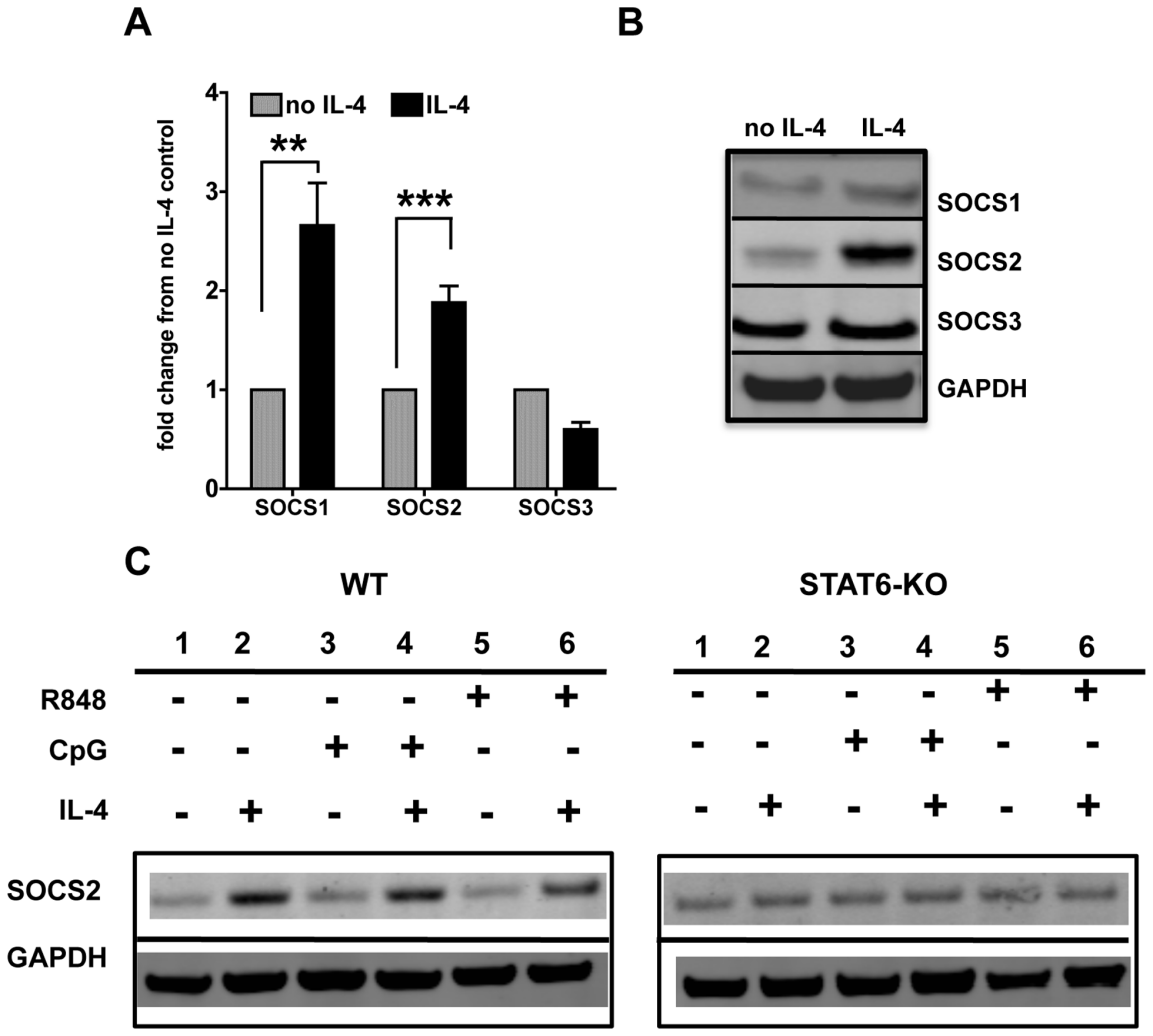
IL-4 induces SOCS1 and SOCS2 gene and protein expression in cDCs. **A.** We analyzed the gene expression of SOCS1, -2 and -3 in cDCs by qPCR after 24 h treatment with IL-4. Results were normalized against untreated cells. Graphs show averages and SE of 6 independent cultures. **B.** We analyzed the protein expression of SOCS2 in the total cDC lysates after 24 h treatment with IL-4. Results were normalized against GAPDH. **C**. We analyzed the protein expression of SOCS2 in wild-type (WT) and STAT6-KO cDCs that had been treated or not with IL-4 for 24 h and then stimulated with CpG or R848 for 8 h. Results were normalized against GAPDH. Blot representative of three sets of experiments.

Suppressors of cytokine signaling (SOCS) molecules are major negative regulators of many cytokine pathways. In particular, SOCS 1 and 3 are known to regulate Type I IFN pathway [32]. We analyzed the gene and protein expression of SOCS1, 2 and 3, after 24 hours of treatment of cDCs with IL-4. We harvested cells for RNA analysis as well as protein analysis by western blot at the same time point to determine whether the gene expression correlated with the protein levels. We observed that both SOCS1 and SOCS2 gene expression levels were increased upon IL-4 treatment, while SOCS3 was slightly decreased (Figure 8A). The gene expression correlated with the protein levels (Figure 8B), with an increase in SOCS1, an even stronger increase in levels of SOCS2 protein but unaltered SOCS3 expression. These results suggest that the SOCS molecules, especially SOCS1 and 2, mediate IL-4 suppressive effects. We further investigated whether SOCS expression was STAT6-dependent (Figure 8C). Indeed, we did not observe any up-regulation of SOCS2 in STAT6-KO cDCs, indicating that SOCS2 protein expression is STAT6-dependent (Figure 8C). In wild-type cDCs, SOCS2 was up-regulated by IL-4 also upon CpG and R848 stimulation, in a similar fashion to that observed with IL-4 alone. This finding suggests that IL-4 alone induces SOCS2, independently from other stimuli, and SOCS2 is likely to be one of the mechanisms of IL-4 suppression of cDC response to TLR7 and TLR9 stimulation. Overall our results indicate that IL-4 induces SOCS1 and SOCS2 molecules that have the ability to regulate TLR7 and TLR9 mediated responses in cDCs.

### IL-4 suppresses IRGs in splenic DCs upon TLR7 and TLR9 stimulation

**Figure 9:**
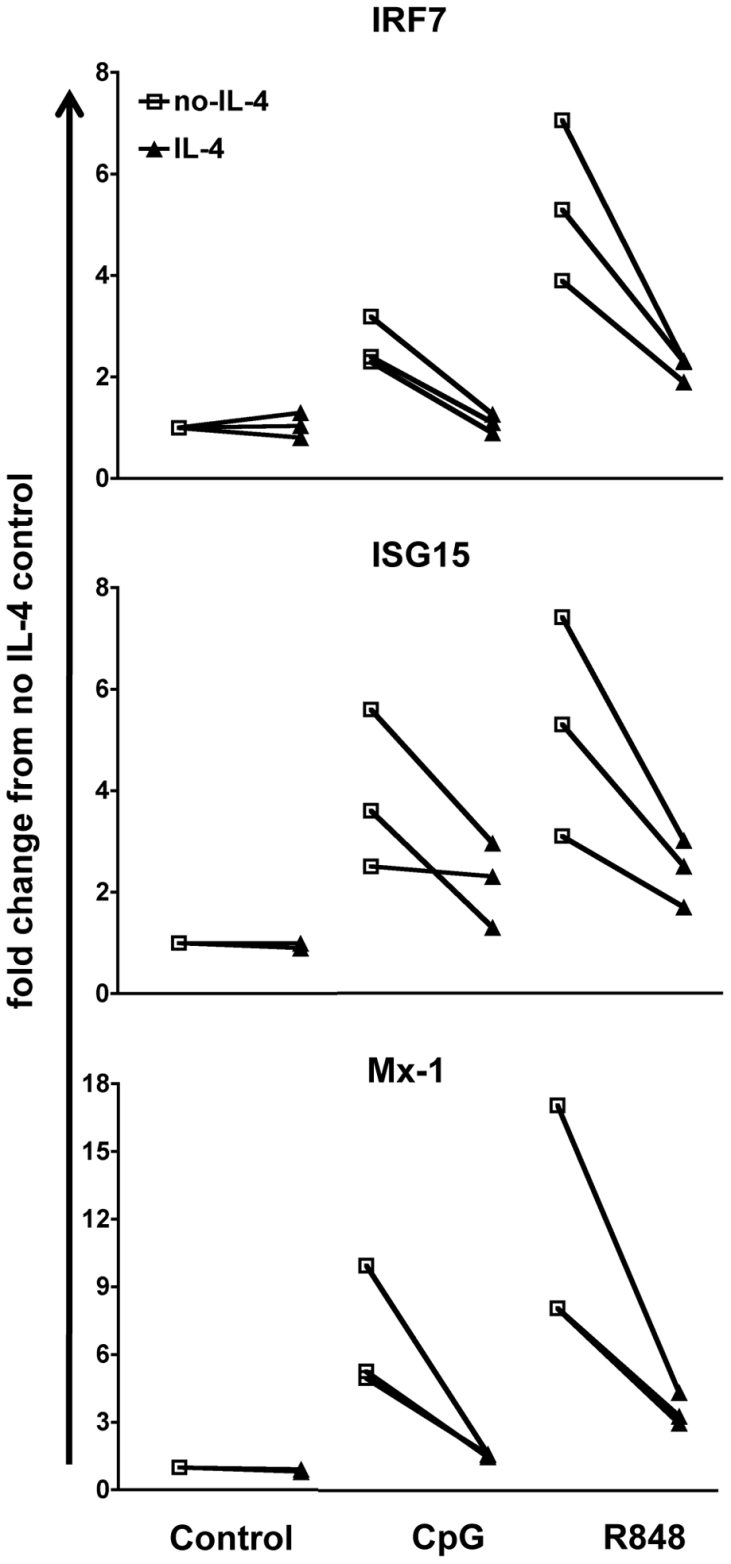
IL-4 suppresses TLR7- and TLR9-induced response in splenic DCs *ex-vivo*. We analyzed the expression of IFN-responsive genes induced by CpG or R848 stimulation in splenic DCs. We cultured the total splenocytes from C57BL/6 mice as 30–50 million cells in 3 ml each well in GM-CSF-complete medium and 2.5 ng/ml of IL-4 overnight and then stimulated with 50 ug CpG or 5 ug R848 for 4 h. Subsequently we sorted the CD11c+ DCs using Miltenyi magnetic bead separation. All the conditions were normalized against the control (GM-CSF medium only) in each experiment. Line graphs show the response calculated as fold change from no IL-4 control of three independent experiments (*p<0.05).

To determine whether IL-4 also affects cDCs that differentiated *in vivo*, we studied splenic DCs. We treated whole spleen cultures with or without IL-4 for approximately 16 hours ex vivo. We then stimulated with CpG or R848 for 4 hours. After sorting cDCs using magnetic beads, we analyzed IRG expression. We found that splenic DCs behaved similarly to bone marrow-derived cDCs in that they up-regulated IRGs IRF7, ISG15 and Mx-1 upon TLR7 and TLR9 stimulation. IL-4 suppressed these effects (Figure 9). This is indicative of a physiological role for IL-4 in cDC response to nucleic acids.

### IL-4 suppresses virus-induced IRG expression and increases permissiveness of cDCs to lentiviral infection

**Figure 10:**
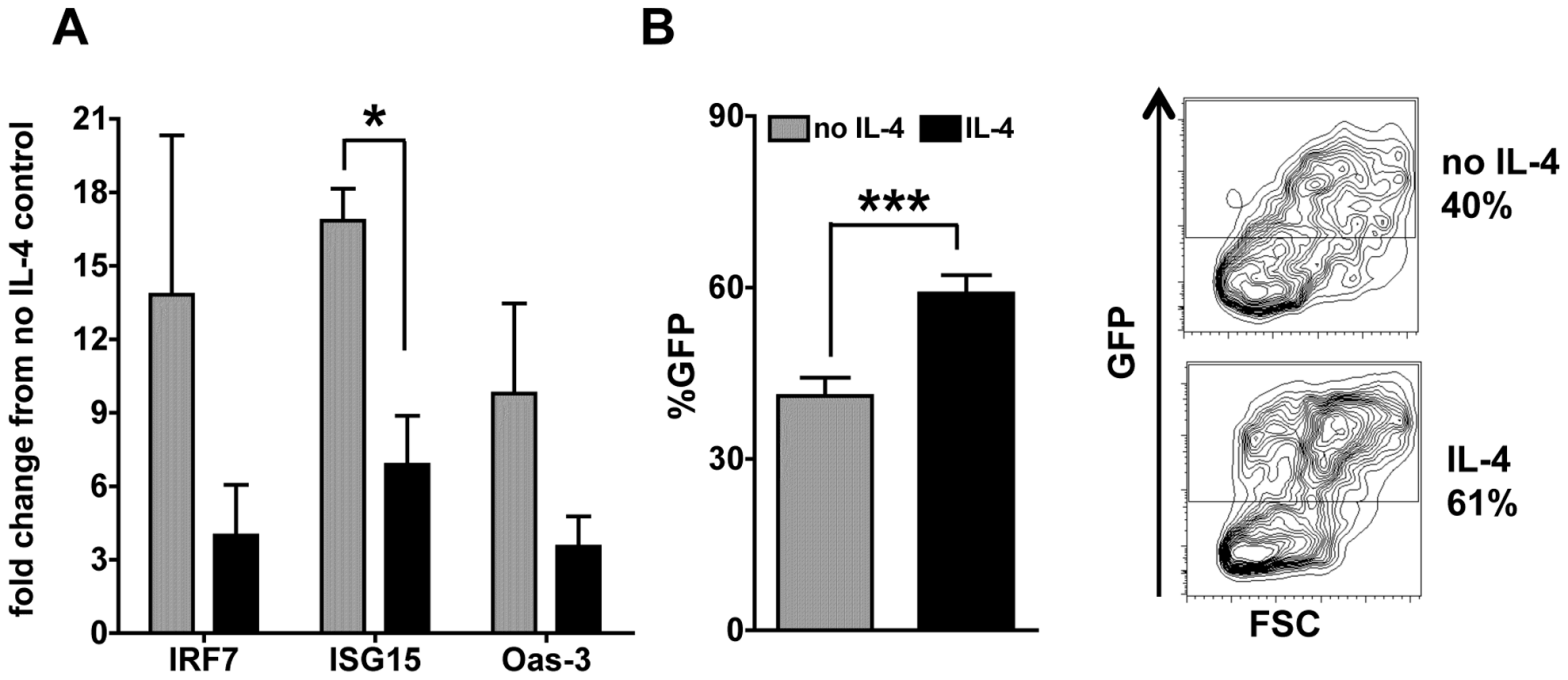
IL-4 decreases virus-induced IRG expression and increases cDC permissiveness to viral infection. **A.** We analyzed the IFN-responsive gene expression after 6 h of viral infection by qPCR. Results were normalized against untreated cells in medium alone. Graphs show averages and SE of two independent experiments tested in duplicate in each experiment. **B.** We measured the GFP fluorescence of cDCs by flow cytometry as a measure of viral infection after 96 h; histograms show the percentage of GFP positive DCs (*left*) and FACS plot show GFP positivity in cells gated for the DC marker CD11c (*right*). Average of two independent cultures tested in duplicate is shown.

TLR7 and TLR9 participate in HIV recognition by innate immune cells, including DCs [49]. Moreover, cDCs constitute an important reservoir for this virus [50], [51]. We used a HIV-based lentivirus [36] carrying GFP as a model to test the effects of IL-4 on the response of cDCs to retroviral infection. cDCs up-regulated IRGs IRF7, ISG15, and Oas-3 6 hours after lentiviral infection and treatment with IL-4 decreased such up-regulation (Figure 10A), mimicking the results that we observed with the synthetic TLR ligands (Figure 1). We also analyzed GFP positivity in cDCs after 96 hours of infection, as a measure of infection and viral expression in the cDCs. We found that the virus was able to infect the cells and successfully express its proteins in an average of 40% of the cDCs, a percentage that was increased to 60% in IL-4-treated cDCs (Figure 10B). These results suggest that IL-4 increases cDC permissiveness to infection by HIV-like retroviruses, possibly by decreasing viral recognition by TLR7/9 and inhibiting the expression of anti-viral IRGs (Figure 10A).

## Discussion

We have shown that IL-4 suppresses cDC responses to stimulation with TLR7 and TLR9 ligands. Indeed IL-4 inhibited IFNβ and IRG expression as well as autocrine Type I IFN signaling as analyzed by the levels of IRF7, IFNAR1 and IFNAR2 genes and STAT1 and STAT2 proteins. IL-4 also inhibited the increase in expression of MHC Class I, another IFN-induced response that is important to efficiently stimulate anti-viral adaptive immune responses. Moreover, IL-4 inhibited the expression and secretion of the classic proinflammatory cytokines IL-12, TNFα and IL-6 upon TLR7 and TLR9 stimulation. Our results are in agreement with Tel *et al.* who have demonstrated that human pDCs exposed to Th2 type environment are impaired in TLR-9-induced secretion of inflammatory cytokines and chemokines [52]. CDCs are pivotal in presenting viral antigens to T cells during viral infections. Therefore, the suppressive effects of IL-4 will have a great impact on the development of the adaptive immune response against viruses [53].

We observed that IL-4 had similar suppressive effects on the splenic DCs as well. The suppression of IRGs was profound in the splenic DCs, as it was in bone marrow-derived DCs, suggesting that IL-4 inhibits not only DCs generated in the isolation of a monocellular culture, but also in the presence of all the other immune cells of the splenic environment.

We have investigated the molecular mechanisms mediating the suppressive effects of IL-4 and found that blocking two major pathways downstream of TLR7/9, Type I IFNs and NF-κB, replicated the IL-4 inhibitory effects on pro-inflammatory cytokines. These results suggest that IL-4 suppression of cDC response to TLR7 and TLR9 stimulation affects both IFN-dependent and NF- κB-dependent responses.

IL-4 mediates most of its effects via STAT6 and STAT6 is pivotal to induce Th2 type response [30]. However, recent studies indicate that some of the responses downstream of IL-4 receptor can bypass STAT6 [54]. Levings and Schrader [54] have shown that STAT6 is required for the IL-4-mediated inhibition of the production of TNFα and IL-12 stimulated by LPS alone. Furthermore, IL-4 also activates distinct, STAT6 independent mechanisms that inhibit the IFNγ-mediated enhancement of IL-12 and TNFα production. Our results indicate that IL-4 inhibition of the expression of both IRGs as well as the pro-inflammatory cytokines induced by TLR7 and TLR9 is STAT6 dependent. Therefore, it was important to understand how STAT6 affects TLR responses. STAT6 could potentially influence the expression of IRG by directly binding to STAT2 and preventing the formation of STAT1-STAT2-IRF9 complex and the activation of the IFN-induced gene expression [55]. Similarly, STAT6 could inhibit the production of pro-inflammatory cytokines by competing with NF-κB DNA-binding activity [56], [57]. Moreover, IL-4 could also inhibit or induce expression of proteins that down-regulate TLR responses [56].

To explore the possibility that IL-4 could inhibit cDC response to CpG and R848 by decreasing the expression of their receptors, we analyzed gene and protein expression of TLR7 and TLR9 upon IL-4 exposure. The interpretation of the results is complicated because IL-4 clearly inhibited the gene of expression of TLR7 and TLR9 while it had no measurable effect on the protein expression of TLR7. The technical difficulties of measuring TLR9 protein expression in un-manipulated primary cultures of cDCs prevent us to conclude anything about the levels of TLR9 proteins. There are many examples in literature in which RNA regulation and protein expression are regulated in different ways[58]. It is cautious to conclude that in the conditions investigated in this project, the suppression of TLR response by IL-4 cannot be explained by a decreased expression of TLRs.

As several reports indicate the role of SOCS molecules as potential negative regulators of IFN [59] and TLR signaling [60], we analyzed the role of SOCS (SOCS1, 2 and 3) and found a strong up-regulation of SOCS1 and especially SOC2 by IL-4, which was STAT6-dependent. Since we did not silence or knockout these molecules, we cannot establish a definitive cause-effect link, but we show a direct correlation between SOCS1 and SOC2 induction and the suppressive effects of IL-4 with either TLR7 or TLR9 stimulations; therefore, we suggest that SOCS2 up-regulation is a likely mechanism for IL-4 inhibitory effects. SOCS1 and SOCS3 have been clearly involved in the regulation of DC activation [61] while few reports have studied the role of SOCS2 in the immune system. It has been previously shown that IL-4 induces SOCS2 in T cells and that SOCS2-/- mice have enhanced Th2 differentiation [62]. Furthermore, SOCS2 has been shown to control macrophage polarization upon exposure to IL-4 [63]. Our suggestion that SOCS2 could mediate IL-4 suppression of TLR7-9 responses in cDCs is also supported by the evidence that SOCS2 can inhibit cytokine production in murine cDCs and human monocyte-derived DCs [31] which are equivalent to the GM-CSF-cDCs that we used. Indeed, Posselt et al. [31] showed that TLR ligands induce a late up-regulation of SOCS2 in cDCs and silencing of SOCS2 lead to increased cytokine production upon TLR stimulation. We did not find up-regulation of SOCS2 by CpG and R848 probably because we looked at early time points, while SOCS2 was consistently up-regulated by IL-4 treatment, irrespective of TLR stimulation, suggesting that it is a dominant mechanism to control DC activation.

IL-4-mediated inhibition of DC responses to TLR7 and TLR9 stimulation can be very important in many immunological reactions and suggests that a Th2 environment modulates the capacity of DCs to respond to endogenous, bacterial- and/or viral-derived nucleic acid sequences [21], [22], [64]. In the present paper, we have analyzed the consequences on the response of DCs to retroviruses. DCs are an important cell type in HIV infection [65]. DCs can become infected at the site of entry of the virus (*cis*-infection) [66] and they participate to the early transmission of HIV to the CD4 T cells (*trans*-infection) [67]. Using a HIV-based-lentivirus as a model of response to HIV-like retroviruses, we found that IL-4 suppresses cDC's ability to produce IFN-responsive genes upon viral recognition and renders cDCs more permissive to the retroviral infection. Since Type I IFNs can restrict both HIV infection of DCs and DC's ability to infect *in trans* CD4 T cells [68], IL-4 suppression of the IFN response of DCs to lentiviruses may affect both pivotal steps in HIV pathogenesis. Our results can provide a molecular explanation for the results of Boyer et al. who have found that IL-4 increases *in vivo* Simian immunodeficiency virus loads in infected rhesus macaques, despite enhanced antibody responses [26]. These findings can be due to the inability of DCs, and possibly other cell types, to respond to the recognition of the virus by TLR7-9. This mechanism can be accompanied by others in cells that do not express TLR7-9: an example is the enhancement of HIV-1 expression in IL-4-producing CD4 T cells by the novel transcription factor c-maf, which is required for IL-4 expression, but also binds to the proximal HIV-1 LTR and augments HIV-1 transcription in partnership with NFAT2 and NF-κB p65 [69].

The HIV-derived lentivirus that we used was originally developed as a vector for gene therapy [37] and tested for expression *in vitro* and *in vivo* [36]. It was found to infect bone marrow-derived cDCs, as measured by its GFP expression [70]. The results that IL-4 increases its expression suggest that gene therapy protocols using lentiviruses are affected by the type of immune response occurring and especially by the Th1/Th2 balance.

Systemic lupus erythematosus is an autoimmune disease in which recognition of endogenous ssRNA and dsDNA by TLR7 and TLR9 is an important pathogenic step for the induction of the IFN Signature and the production of autoantibodies against many self-antigens, including dsDNA [5], [71]. Our results suggest that IL-4 could inhibit the TLR7-9 pathogenic steps and decrease the production of the IFN Signature in lupus [18].

In conclusion, our results indicate that IL-4 inhibits the response of cDCs to TLR7 and TLR9 stimulation via STAT6 signaling. This suppression can contribute to the inhibition of the immune responses to viral infections established by a Th2 environment. IL-4 suppressive effects on IFN response could play a role in the spreading of HIV in DCs, a natural reservoir for this retrovirus, during chronic Th2 conditions like helminth infestation in sub-Saharan Africa. Moreover, IL-4 effects should be considered during the design of gene therapy protocols utilizing lentiviral vectors. Finally, IL-4 may be beneficial in blocking the recognition of self-adjuvants and self-antigens in systemic lupus erythematosus.

## Supporting Information

Figure S1 **IL-4 suppresses the TLR7- and TLR9-induced IFNβ gene expression.** We analyzed by qPCR the expression of IFNβ in cDCs treated or not with IL-4 for 24 h and after 6 h of CpG or R848 stimulation. All of the conditions were normalized against the control (untreated DCs in medium only) in each experiment. Results are average of three independent experiments.
(TIFF)

Figure S2 **IL-4 suppression of pro-inflammatory cytokines is not dependent on IFNAR or NF-κB.** We measured by ELISA the levels of IL-6, IL-12p70 and TNFα in the supernatants of cDC cultures from wild type and IFNAR1-KO mice treated or not with IL-4 and stimulated with CpG 1826 or R848 for 24 h. Bay11 (10^−6^ M) was added in some wells 20 min before TLR induction as an NF-κB inhibitor. Averages and STDEV of two independent cultures are shown.
(TIFF)

Figure S3 **IL-4 suppresses IFNAR1 and IFNAR2 gene expression in cDCs.** We analyzed the expression of IFNAR1 and IFNAR2 genes by qPCR in the RAG-KO cDCs after 24 h treatment with IL-4. All of the conditions were normalized against the control (untreated DCs in medium only) in each experiment. Results are mean and SE of four independent experiments.
(TIFF)

Figure S4 **IL-4 suppresses TLR7 and TLR9 gene expression induced by CpG or R848 in cDCs.** We analyzed the gene expression of TLR7 and TLR9 by qPCR in the RAG-KO cDCs after 24 h treatment with IL-4 and then stimulated with CpG or R848 for 6 h. All of the conditions were normalized against the control (untreated DCs in medium only) in each experiment. Results are mean and SE of three independent experiments.
(TIFF)
