# Supplementary figures and images for: IL-4 Suppresses the Responses to TLR7 and TLR9 Stimulation and Increases the Permissiveness to Retroviral Infection of Murine Conventional Dendritic Cells
Source: PLoS One. 2014 Jan 29;9(1):e87668. doi: 10.1371/journal.pone.0087668 (PMC3906189; doi:10.1371/journal.pone.0087668)

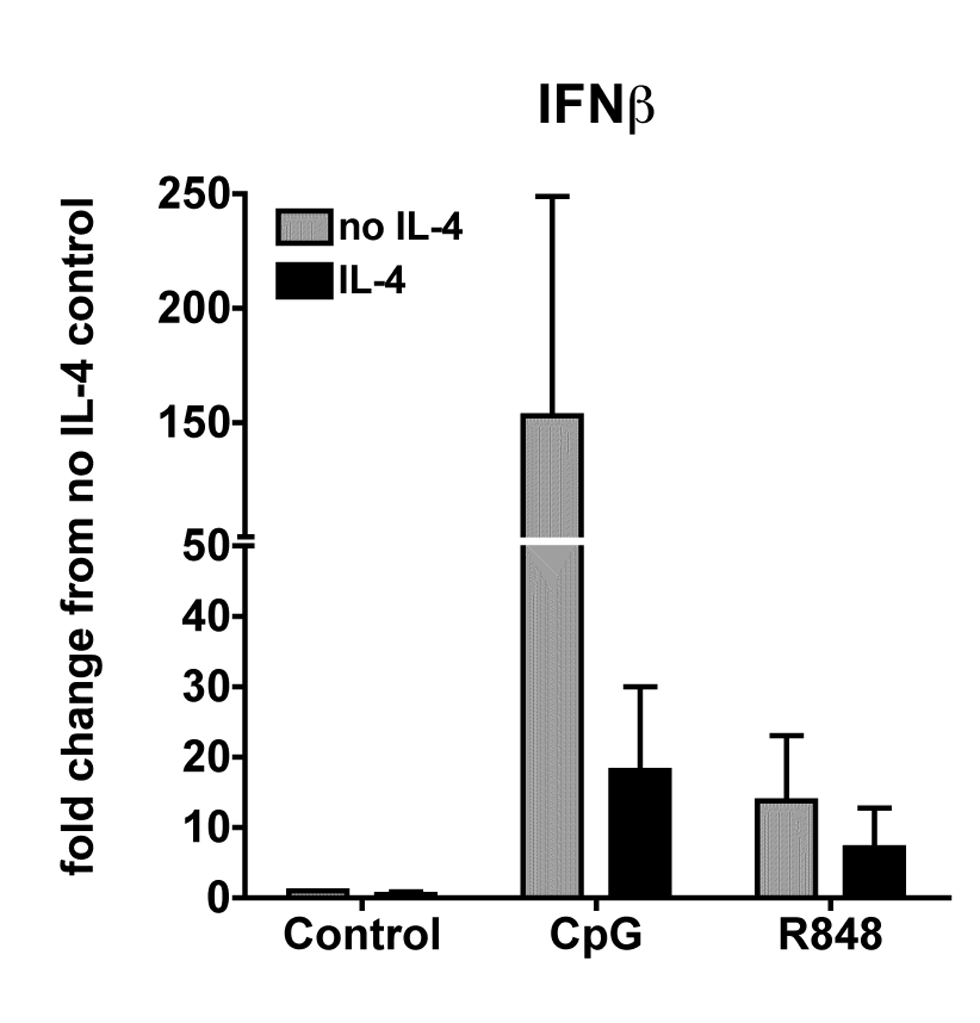

Supplement: Figure S1 — IL-4 suppresses the TLR7- and TLR9-induced IFNβ gene expression. We analyzed by qPCR the expression of IFNβ in cDCs treated or not with IL-4 for 24 h and after 6 h of CpG or R848 stimulation. All of the conditions were normalized against the control (untreated DCs in medium only) in each experiment. Results are average of three independent experiments. (TIFF) [file pone.0087668.s001.tiff]

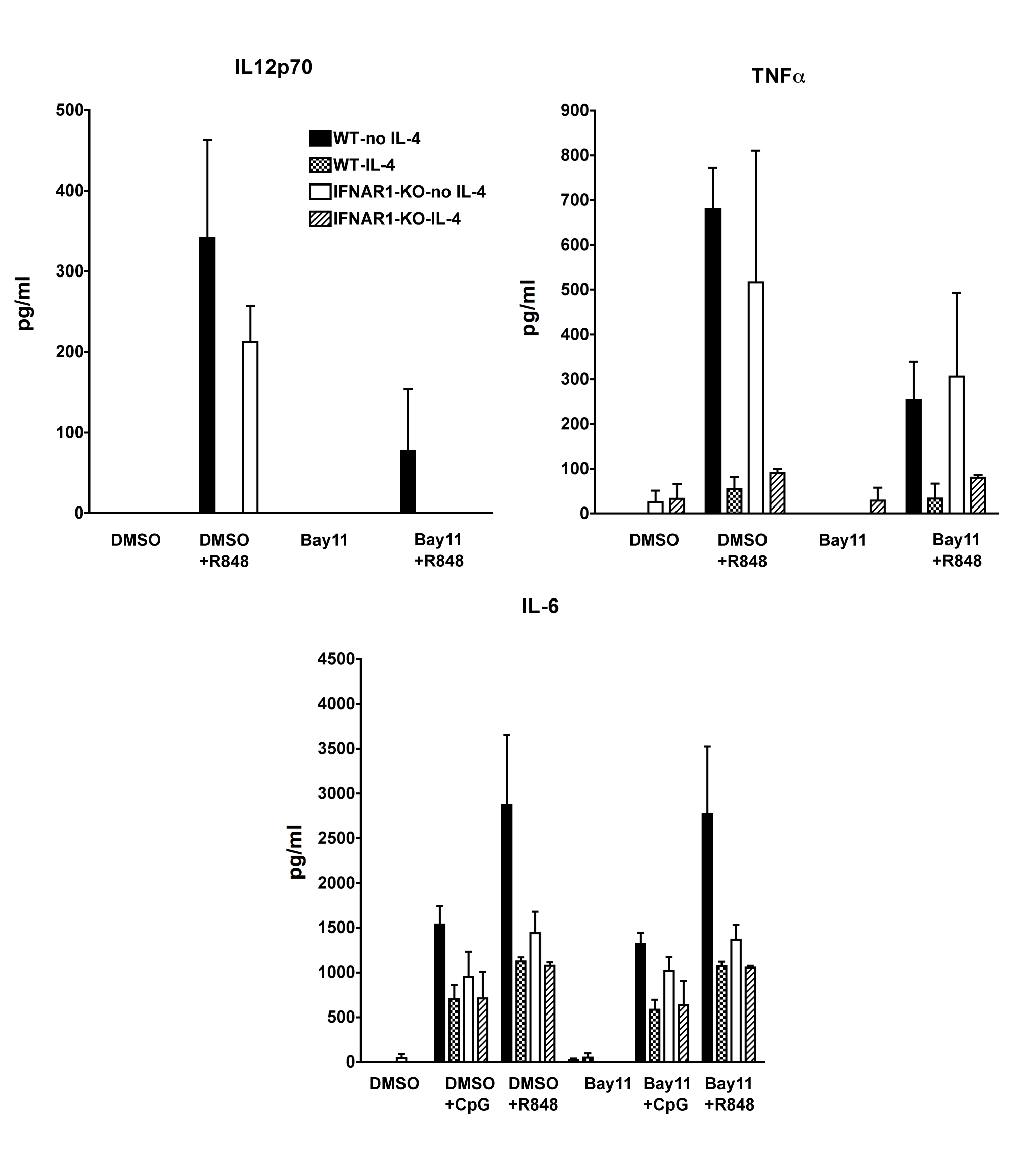

Supplement: Figure S2 — IL-4 suppression of pro-inflammatory cytokines is not dependent on IFNAR or NF-κB. We measured by ELISA the levels of IL-6, IL-12p70 and TNFα in the supernatants of cDC cultures from wild type and IFNAR1-KO mice treated or not with IL-4 and stimulated with CpG 1826 or R848 for 24 h. Bay11 (10−6 M) was added in some wells 20 min before TLR induction as an NF-κB inhibitor. Averages and STDEV of two independent cultures are shown. (TIFF) [file pone.0087668.s002.tiff]

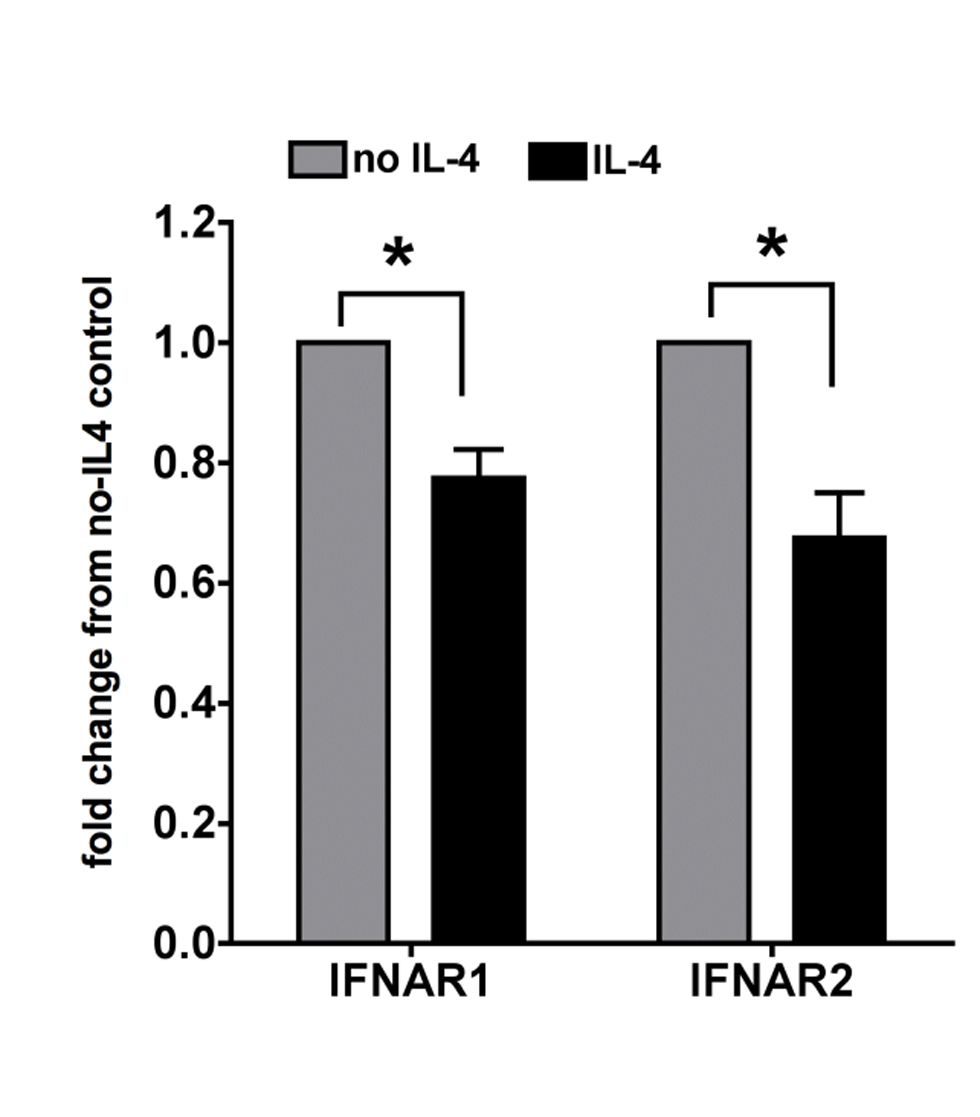

Supplement: Figure S3 — IL-4 suppresses IFNAR1 and IFNAR2 gene expression in cDCs. We analyzed the expression of IFNAR1 and IFNAR2 genes by qPCR in the RAG-KO cDCs after 24 h treatment with IL-4. All of the conditions were normalized against the control (untreated DCs in medium only) in each experiment. Results are mean and SE of four independent experiments. (TIFF) [file pone.0087668.s003.tiff]

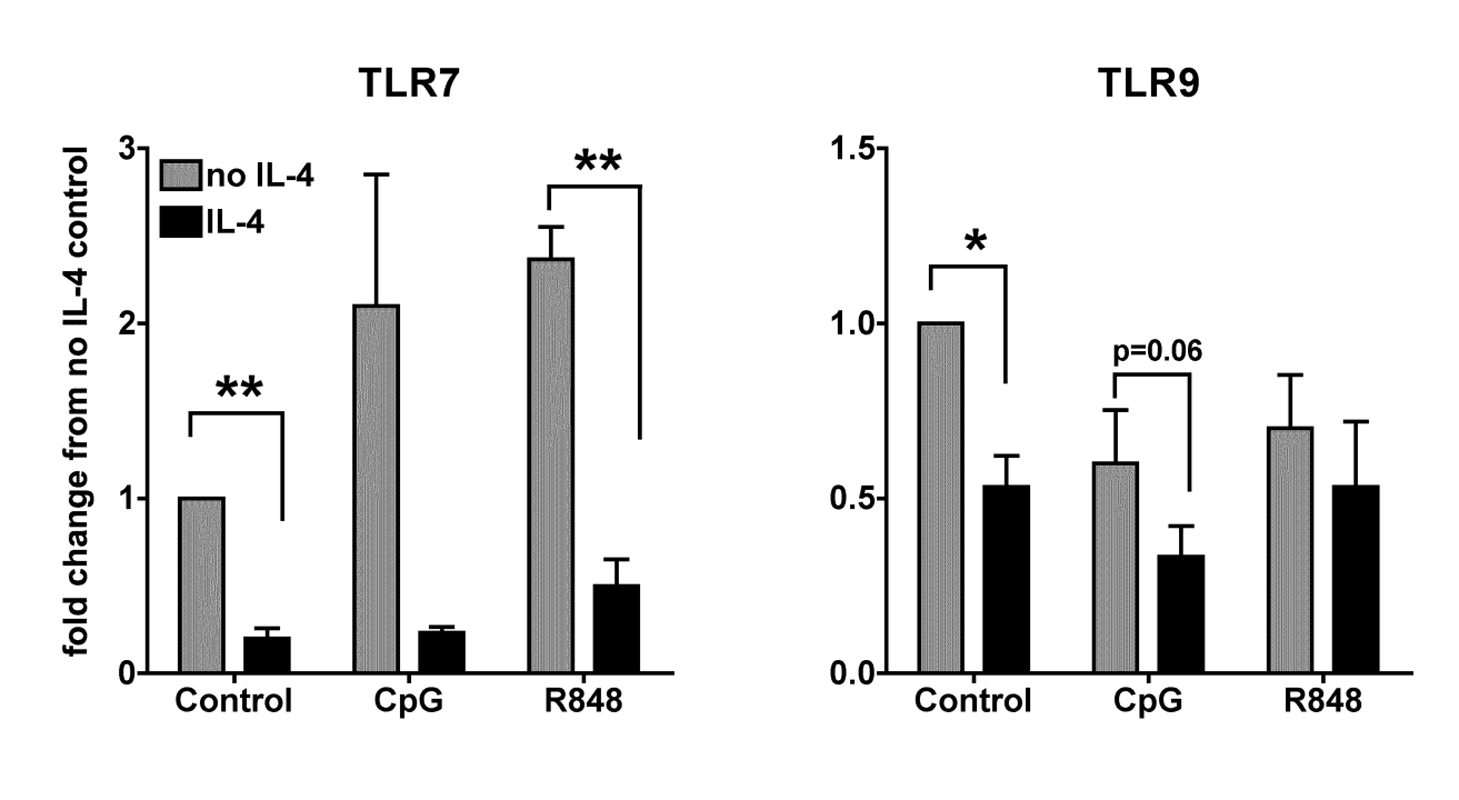

Supplement: Figure S4 — IL-4 suppresses TLR7 and TLR9 gene expression induced by CpG or R848 in cDCs. We analyzed the gene expression of TLR7 and TLR9 by qPCR in the RAG-KO cDCs after 24 h treatment with IL-4 and then stimulated with CpG or R848 for 6 h. All of the conditions were normalized against the control (untreated DCs in medium only) in each experiment. Results are mean and SE of three independent experiments. (TIFF) [file pone.0087668.s004.tiff]
